# 2-(Eth­oxy­carbon­yl)pyridinium nitrate

**DOI:** 10.1107/S1600536810025407

**Published:** 2010-07-14

**Authors:** Yingchun Wang

**Affiliations:** a Ordered Matter Science Research Center, College of Chemistry and Chemical Engineering, Southeast University, Nanjing 210096, People’s Republic of China

## Abstract

In the title compound, C_8_H_10_NO_2_^+^·NO_3_^−^, the cation is essentially planar with C—O—C—C and C—O—C—O torsion angles of −178.1 (2) and 2.1 (4)°, respectively. In the crystal, N—H⋯O and C—H⋯O hydrogen-bond inter­actions stabilize the structure.

## Related literature

For phase transition of pyridinium salts studied by X-ray analysis and dielectric and heat capacity measurements, see: Asaji *et al.* (2007 ▶). For their ferroelecric properties, see: Wasicki *et al.* (1997 ▶).
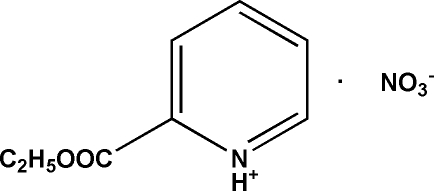


## Experimental

### 

#### Crystal data

C_8_H_10_NO_2_^+^·NO_3_^−^
*M* *_r_* = 214.18
Monoclinic, 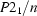

*a* = 6.8221 (14) Å
*b* = 16.208 (3) Å
*c* = 9.2195 (18) Å
β = 106.55 (3)°
*V* = 977.2 (3) Å^3^
*Z* = 4
Mo *K*α radiation
μ = 0.12 mm^−1^
*T* = 293 K
0.20 × 0.20 × 0.20 mm

#### Data collection

Rigaku SCXmini diffractometer
Absorption correction: multi-scan (*CrystalClear*; Rigaku, 2005 ▶) *T*_min_ = 0.976, *T*_max_ = 0.976
9694 measured reflections
2226 independent reflections
1287 reflections with *I* > 2σ(*I*)
*R*_int_ = 0.089

#### Refinement

*R*[*F* ^2^ > 2σ(*F* ^2^)] = 0.064
*wR*(*F* ^2^) = 0.178
*S* = 1.04
2226 reflections
136 parameters
H-atom parameters constrained
Δρ_max_ = 0.22 e Å^−3^
Δρ_min_ = −0.22 e Å^−3^

### 

Data collection: *CrystalClear* (Rigaku, 2005 ▶); cell refinement: *CrystalClear*; data reduction: *CrystalClear*; program(s) used to solve structure: *SHELXS97* (Sheldrick, 2008 ▶); program(s) used to refine structure: *SHELXL97* (Sheldrick, 2008 ▶); molecular graphics: *SHELXTL* (Sheldrick, 2008 ▶); software used to prepare material for publication: *SHELXL97*.

## Supplementary Material

Crystal structure: contains datablocks I, global. DOI: 10.1107/S1600536810025407/fl2300sup1.cif

Structure factors: contains datablocks I. DOI: 10.1107/S1600536810025407/fl2300Isup2.hkl

Additional supplementary materials:  crystallographic information; 3D view; checkCIF report

## Figures and Tables

**Table 1:** Hydrogen-bond geometry (Å, °)

*D*—H⋯*A*	*D*—H	H⋯*A*	*D*⋯*A*	*D*—H⋯*A*
N1—H1*A*⋯O4	0.86	1.91	2.759 (3)	170
C1—H1*B*⋯O3	0.93	2.38	3.078 (4)	131
C8—H8*A*⋯O3^i^	0.96	2.57	3.506 (4)	166

Symmetry code: (i) 
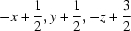
.

**Fig. 1.**
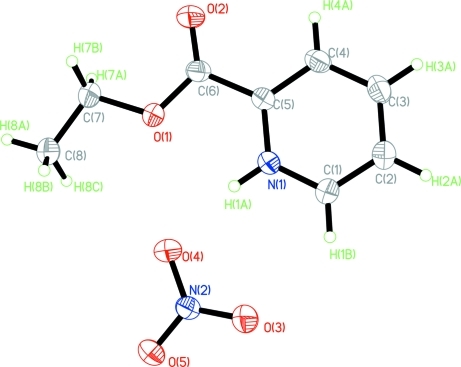
The molecular structure of the title compound with the atomic numbering scheme. Displacement ellipsoids are drawn at the 30% probability level, and all H atoms have been omitted for clarity.

**Fig. 2.**
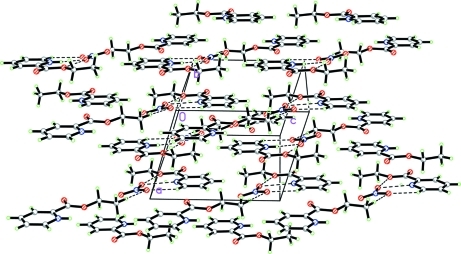
A view of the packing of the title compound, stacking along the b axis. Dashed lines indicate hydrogen bonds.

## supplementary crystallographic information

### Comment

The study of seignette-electrics materials has received much attention. Some materials exhibit predominant dielectric-ferroelectric properties such as pyridine single salts of the PyHX type (*X*=ICl_4_, ClO_4_, IO_4_, ReO_4, _*etc*.) ( Asaji *et al.*(2007); Wasicki *et al.* (1997)). As one part of our continuing studies on looking for materials with these properties, we have used 2-ethyl picolinate as the ligand and synthesized salts similar to PyHX. The title compound (I) is one of these salts. It exhibits no phase-transition in dielectric measurement going from 93 K to 340 K (m.p 348 K).

The asymmetric unit of (I) contains one picolinate cation and one nitrate radical (Fig 1). The pyridine ring is planar and the carbethoxy is in the plane of the ring with an O2—C6—C5—C4 torsion angle of 0.1 (4)°. The torsion angles C7—O1—C6—C5 and C7—O1—C6—O2 at -178.1 (2)° and 2.1 (4)° respectively also show the overall planarity of the cation. Intramolecular N1—H···O4 and C1—H1B···O3 interactions link the cation and the anion while intermolecular C8—H8A···O3 interactions link the molecules into chains (Table 1, Fig 2).

### Experimental

A solution of 2-ethyl picolinate (10 mmol) in ethanol (20 ml) was added to a solution of equimolar amount of aqua fortis aqueous solution (1 mol/*L*). Crystals suitable for structure determination were grown by slow evaporation of the mixture at room temperature.

### Refinement

Positional parameters of all the H atoms were calculated geometrically and were allowed to ride on the C atoms to which they are bonded, with C—H = 0.93 Å, N—H = 0.75–0.86 Å; with *U*_iso_(H) = 1.2Ueq(C), with *U*_iso_(H) = 1.2–1.5Ueq(N).

### Crystal data

**Table:**

C_8_H_10_NO_2_^+^·NO_3_^−^	*F*(000) = 448
*M**_r_* = 214.18	*D*_x_ = 1.456 Mg m^−^^3^
Monoclinic, *P*2_1_/*n*	Mo *K*α radiation, λ = 0.71073 Å
Hall symbol: -P 2yn	Cell parameters from 3542 reflections
*a* = 6.8221 (14) Å	θ = 3.1–27.6°
*b* = 16.208 (3) Å	µ = 0.12 mm^−^^1^
*c* = 9.2195 (18) Å	*T* = 293 K
β = 106.55 (3)°	Prism, colourless
*V* = 977.2 (3) Å^3^	0.20 × 0.20 × 0.20 mm
*Z* = 4	

### Data collection

**Table:**

Rigaku SCXmini diffractometer	2226 independent reflections
Radiation source: fine-focus sealed tube	1287 reflections with *I* > 2σ(*I*)
graphite	*R*_int_ = 0.089
Detector resolution: 13.6612 pixels mm^-1^	θ_max_ = 27.5°, θ_min_ = 3.3°
ω scans	*h* = −8→8
Absorption correction: multi-scan (*CrystalClear*; Rigaku, 2005)	*k* = −21→21
*T*_min_ = 0.976, *T*_max_ = 0.976	*l* = −11→11
9694 measured reflections	

### Refinement

**Table:**

Refinement on *F*^2^	Primary atom site location: structure-invariant direct methods
Least-squares matrix: full	Secondary atom site location: difference Fourier map
*R*[*F*^2^ > 2σ(*F*^2^)] = 0.064	Hydrogen site location: inferred from neighbouring sites
*wR*(*F*^2^) = 0.178	H-atom parameters constrained
*S* = 1.04	*w* = 1/[σ^2^(*F*_o_^2^) + (0.072*P*)^2^ + 0.2351*P*] where *P* = (*F*_o_^2^ + 2*F*_c_^2^)/3
2226 reflections	(Δ/σ)_max_ < 0.001
136 parameters	Δρ_max_ = 0.22 e Å^−^^3^
0 restraints	Δρ_min_ = −0.21 e Å^−^^3^

### Special details

**Table:**

Geometry. All e.s.d.'s (except the e.s.d. in the dihedral angle between two l.s. planes) are estimated using the full covariance matrix. The cell e.s.d.'s are taken into account individually in the estimation of e.s.d.'s in distances, angles and torsion angles; correlations between e.s.d.'s in cell parameters are only used when they are defined by crystal symmetry. An approximate (isotropic) treatment of cell e.s.d.'s is used for estimating e.s.d.'s involving l.s. planes.
Refinement. Refinement of *F*^2^ against ALL reflections. The weighted *R*-factor *wR* and goodness of fit *S* are based on *F*^2^, conventional *R*-factors *R* are based on *F*, with *F* set to zero for negative *F*^2^. The threshold expression of *F*^2^ > σ(*F*^2^) is used only for calculating *R*-factors(gt) *etc*. and is not relevant to the choice of reflections for refinement. *R*-factors based on *F*^2^ are statistically about twice as large as those based on *F*, and *R*- factors based on ALL data will be even larger.

### Fractional atomic coordinates and isotropic or equivalent isotropic displacement parameters (Å^2^)

**Table:**

	*x*	*y*	*z*	*U*_iso_*/*U*_eq_	
O1	0.2450 (3)	0.15158 (11)	0.4900 (2)	0.0500 (6)	
O5	0.1957 (4)	−0.10679 (14)	0.7615 (2)	0.0641 (7)	
N1	0.2593 (3)	0.01904 (14)	0.3286 (2)	0.0410 (6)	
H1A	0.2558	0.0168	0.4210	0.049*	
O4	0.2397 (4)	−0.00834 (13)	0.6195 (3)	0.0730 (8)	
O3	0.2805 (4)	−0.13303 (14)	0.5588 (2)	0.0727 (7)	
N2	0.2370 (3)	−0.08353 (15)	0.6463 (3)	0.0445 (6)	
C6	0.2474 (4)	0.16983 (18)	0.3508 (3)	0.0445 (7)	
C5	0.2573 (4)	0.09338 (17)	0.2627 (3)	0.0406 (7)	
O2	0.2430 (3)	0.23734 (13)	0.2988 (2)	0.0611 (7)	
C3	0.2670 (5)	0.0251 (2)	0.0360 (3)	0.0540 (8)	
H3A	0.2693	0.0270	−0.0643	0.065*	
C4	0.2621 (4)	0.09761 (19)	0.1144 (3)	0.0503 (8)	
H4A	0.2621	0.1485	0.0677	0.060*	
C1	0.2664 (4)	−0.05092 (18)	0.2550 (3)	0.0472 (8)	
H1B	0.2701	−0.1012	0.3043	0.057*	
C7	0.2289 (5)	0.22041 (19)	0.5875 (3)	0.0567 (9)	
H7A	0.1064	0.2522	0.5417	0.068*	
H7B	0.3465	0.2565	0.6026	0.068*	
C2	0.2684 (5)	−0.04929 (19)	0.1065 (4)	0.0513 (8)	
H2A	0.2707	−0.0982	0.0544	0.062*	
C8	0.2201 (6)	0.1856 (2)	0.7339 (4)	0.0611 (9)	
H8A	0.2077	0.2297	0.8004	0.092*	
H8B	0.3429	0.1550	0.7788	0.092*	
H8C	0.1040	0.1497	0.7175	0.092*	

### Atomic displacement parameters (Å^2^)

**Table:**

	*U*^11^	*U*^22^	*U*^33^	*U*^12^	*U*^13^	*U*^23^
O1	0.0731 (15)	0.0370 (11)	0.0431 (11)	−0.0019 (9)	0.0217 (10)	−0.0039 (9)
O5	0.0931 (17)	0.0609 (14)	0.0470 (12)	−0.0035 (12)	0.0340 (12)	0.0060 (10)
N1	0.0460 (14)	0.0425 (14)	0.0376 (12)	−0.0003 (11)	0.0172 (11)	0.0002 (10)
O4	0.124 (2)	0.0409 (13)	0.0617 (14)	−0.0030 (12)	0.0388 (14)	0.0056 (11)
O3	0.116 (2)	0.0547 (14)	0.0573 (14)	0.0149 (13)	0.0415 (14)	0.0021 (12)
N2	0.0493 (15)	0.0443 (15)	0.0406 (14)	−0.0017 (11)	0.0138 (11)	0.0039 (11)
C6	0.0479 (18)	0.0420 (16)	0.0449 (16)	−0.0037 (13)	0.0154 (14)	0.0046 (13)
C5	0.0422 (16)	0.0431 (17)	0.0384 (15)	−0.0007 (12)	0.0145 (12)	0.0033 (12)
O2	0.0861 (17)	0.0428 (13)	0.0580 (14)	0.0003 (11)	0.0261 (12)	0.0106 (10)
C3	0.059 (2)	0.066 (2)	0.0412 (17)	−0.0029 (16)	0.0199 (15)	−0.0040 (15)
C4	0.058 (2)	0.0517 (18)	0.0442 (16)	−0.0006 (15)	0.0188 (14)	0.0087 (14)
C1	0.0542 (19)	0.0420 (18)	0.0474 (17)	0.0005 (13)	0.0176 (14)	−0.0042 (13)
C7	0.082 (2)	0.0418 (17)	0.0463 (18)	−0.0006 (16)	0.0181 (16)	−0.0061 (14)
C2	0.053 (2)	0.0539 (19)	0.0464 (17)	0.0032 (15)	0.0139 (14)	−0.0073 (14)
C8	0.086 (2)	0.0526 (19)	0.0507 (18)	−0.0035 (17)	0.0289 (17)	−0.0091 (15)

### Geometric parameters (Å, °)

**Table:**

O1—C6	1.321 (3)	C3—C4	1.385 (4)
O1—C7	1.457 (3)	C3—H3A	0.9300
O5—N2	1.233 (3)	C4—H4A	0.9300
N1—C1	1.329 (3)	C1—C2	1.373 (4)
N1—C5	1.348 (3)	C1—H1B	0.9300
N1—H1A	0.8600	C7—C8	1.480 (4)
O4—N2	1.245 (3)	C7—H7A	0.9700
O3—N2	1.232 (3)	C7—H7B	0.9700
C6—O2	1.192 (3)	C2—H2A	0.9300
C6—C5	1.494 (4)	C8—H8A	0.9600
C5—C4	1.378 (4)	C8—H8B	0.9600
C3—C2	1.369 (4)	C8—H8C	0.9600
			
C6—O1—C7	116.9 (2)	N1—C1—C2	120.3 (3)
C1—N1—C5	122.0 (2)	N1—C1—H1B	119.8
C1—N1—H1A	119.0	C2—C1—H1B	119.8
C5—N1—H1A	119.0	O1—C7—C8	107.5 (2)
O3—N2—O5	121.4 (2)	O1—C7—H7A	110.2
O3—N2—O4	119.2 (2)	C8—C7—H7A	110.2
O5—N2—O4	119.4 (2)	O1—C7—H7B	110.2
O2—C6—O1	126.2 (3)	C8—C7—H7B	110.2
O2—C6—C5	122.9 (3)	H7A—C7—H7B	108.5
O1—C6—C5	110.9 (2)	C3—C2—C1	119.3 (3)
N1—C5—C4	119.4 (3)	C3—C2—H2A	120.3
N1—C5—C6	119.5 (2)	C1—C2—H2A	120.3
C4—C5—C6	121.0 (2)	C7—C8—H8A	109.5
C2—C3—C4	119.8 (3)	C7—C8—H8B	109.5
C2—C3—H3A	120.1	H8A—C8—H8B	109.5
C4—C3—H3A	120.1	C7—C8—H8C	109.5
C5—C4—C3	119.1 (3)	H8A—C8—H8C	109.5
C5—C4—H4A	120.4	H8B—C8—H8C	109.5
C3—C4—H4A	120.4		
			
C7—O1—C6—O2	2.1 (4)	N1—C5—C4—C3	0.6 (4)
C7—O1—C6—C5	−178.1 (2)	C6—C5—C4—C3	−178.7 (3)
C1—N1—C5—C4	0.2 (4)	C2—C3—C4—C5	−0.4 (4)
C1—N1—C5—C6	179.5 (3)	C5—N1—C1—C2	−1.2 (4)
O2—C6—C5—N1	−179.2 (3)	C6—O1—C7—C8	177.6 (3)
O1—C6—C5—N1	1.0 (4)	C4—C3—C2—C1	−0.5 (5)
O2—C6—C5—C4	0.1 (4)	N1—C1—C2—C3	1.3 (4)
O1—C6—C5—C4	−179.8 (2)		

### Hydrogen-bond geometry (Å, °)

**Table:**

*D*—H···*A*	*D*—H	H···*A*	*D*···*A*	*D*—H···*A*
N1—H1A···O4	0.86	1.91	2.759 (3)	170
C1—H1B···O3	0.93	2.38	3.078 (4)	131
C8—H8A···O3^i^	0.96	2.57	3.506 (4)	166

Symmetry codes: (i) −*x*+1/2, *y*+1/2, −*z*+3/2.
